# Tirzepatide—Friend or Foe in Diabetic Cancer Patients?

**DOI:** 10.3390/biom12111580

**Published:** 2022-10-28

**Authors:** Samson Mathews Samuel, Elizabeth Varghese, Peter Kubatka, Dietrich Büsselberg

**Affiliations:** 1 Department of Physiology and Biophysics, Weill Cornell Medicine-Qatar, Education City, Qatar Foundation, Doha 24144, Qatar; 2 Department of Medical Biology, Jessenius Faculty of Medicine, Comenius University in Bratislava, 03601 Martin, Slovakia

**Keywords:** cancer, diabetes, glycemic control, hyperglycemia, LY3298176, medullary thyroid cancer/carcinoma, tirzepatide

## Abstract

It is a well-accepted fact that obesity and diabetes increase the risk of incidence of different cancers and their progression, leading to a decrease in the quality of life among affected cancer patients. In addition to decreasing the risk of cancers, maintaining a healthy body mass index (BMI)/body weight and/or blood glucose levels within the normal range critically impacts the response to anti-cancer therapy among affected individuals. A cancer patient managing their body weight and maintaining blood glucose control responds better to anti-cancer therapy than obese individuals and those whose blood glucose levels remain higher than normal during therapeutic intervention. In some cases, anti-diabetic/glucose-lowering drugs, some of which are also used to promote weight loss, were found to possess anti-cancer potential themselves and/or support anti-cancer therapy when used to treat such patients. On the other hand, certain glucose-lowering drugs promoted the cancer phenotype and risked cancer progression when used for treatment. Tirzepatide (TRZD), the glucagon-like peptide 1 (GLP-1) and glucose-dependent insulinotropic polypeptide/gastric inhibitory peptide (GIP) agonist, has recently gained interest as a promising injectable drug for the treatment of type 2 diabetes and was approved by the FDA after successful clinical trials (SURPASS 1/2/3/4 and 5, NCT03954834, NCT03987919, NCT03882970, NCT03730662, and NCT04039503). In addition, the reports from the SURMOUNT-1 clinical trial (NCT04184622) support the use of TRZD as an anti-obesity drug. In the current review article, we examine the possibility and molecular mechanisms of how TRZD intervention could benefit cancer therapeutics or increase the risk of cancer progression when used as an anti-diabetic drug in diabetic patients.

## 1. Incretin Mimetics for the Treatment of Type 2 Diabetes

Obesity and type 2 diabetes (hereafter mentioned as diabetes, unless otherwise specified) are among the most common chronic diseases worldwide, affecting all cross-sections of society, including children, women, youth, and adults. They are the leading causes of morbidity and mortality in the modern world [1,2,3,4]. Although the data from the international diabetes federation (IDF) projects a rise of about 10.9% in global diabetes prevalence by 2045, the obesity prevalence has increased in pandemic proportions over the past five decades [5,6]. Apart from the usual obesity and/or diabetes-related co-morbid complications, such as cardio- and cerebro-vascular diseases, hypertension, liver and renal diseases, and infertility, both obesity and diabetes are closely linked to the incidence, progression, and aggressiveness of many different cancers (breast, ovarian, pancreatic, liver, kidney, and colorectal cancers) [1,6,7,8]. This is partly correlated to the metabolic alterations, pro-inflammatory state, and subdued immune responses characteristic of obesity and diabetes [1,7,8].

The link between diabetes and cancer and the fact that cancer cells are essentially dependent on glucose (glycolysis; Warburg effect; to sustain their rapid rate of growth and proliferation) generated interest in studying the effects of widely prescribed anti-hyperglycemic/anti-diabetic drugs on the risk, incidence, progression, response to therapy, resistance and post-therapeutic relapse in cancers [9]. In addition to the blood glucose-lowering effect of these anti-hyperglycemic drugs (and thus restricting glucose availability to cancer cells), some of the tested anti-hyperglycemic drugs (e.g., metformin) have an anti-cancer potential via the inhibition of several different cancer-promoting pathways and by the activation of pathways that cause cancer cell death [8,9]. Coincidentally, many of these glucose-lowering medications or anti-diabetic drugs (metformin, SGLT2 inhibitors, GLP-1 receptor agonists, DPP4 inhibitors) were also effective for the treatment of obesity and for promoting and maintaining weight loss in both diabetic and non-diabetic individuals, albeit when supported by lifestyle changes, a balanced diet, and exercise [10,11,12,13,14,15,16]. In contrast, anti-hyperglycemic interventions such as insulin, thiazolidinediones, and sulphonylureas promote weight gain [17,18,19].

In May 2022, Tirzepatide (TRZD), marketed as Mounjaro (Eli Lilly and Company, Indianapolis, IN, USA), was approved by the Food and Drug Administration (FDA, Silver Spring, MD, USA) as an anti-hyperglycemic drug that improves glycemic control in adults with diabetes. TRZD, an incretin mimetic class of anti-diabetic drugs, is unique as it is the first and only dual-acting (glucose-dependent insulinotropic polypeptide/gastric inhibitory peptide/GIP and glucagon-like peptide 1 receptor/GLP-1R agonist) injectable anti-hyperglycemic drug showing significant improvements in glycemic control and body weight without the apparent risk of hypoglycemia [20]. Additionally, administration of the anti-hyperglycemic dosage of TRZD (5 mg, 10 mg, and 15 mg TRZD once weekly) was associated with a significant and sustained reduction in body weight [21].

Interestingly, incretin (GLP-1 and GIP) mimetics/agonists are known to cause (1) pancreatic damage (pancreatitis) and possible malignant transformation of the pancreas; and (2) promote the development of medullary thyroid carcinoma (MTC) in mice and possibly in patients with a family history of MTC, and (3) thyroid cancers in patients having multiple endocrine neoplasia syndrome type 2 (MEN2) [22,23,24]. TRZD administration, being a dual-acting incretin mimetic, is thus not recommended (by Eli Lilly and Company in their press release) in patients who have had pancreatitis and should not be used in patients with MTC (or anyone in the family of the patient who has had MTC) or if the patient has multiple MEN2 [25].

Apart from these cautionary suggestions/recommendations, there is little direct scientific evidence to suggest that there is reason to exercise caution regarding TRZD administration, possibly concerning cancer (pancreatic and thyroid) incidence and progression. Additionally, it remains unknown whether TRZD, indirectly via its blood glucose-lowering effect or directly by modulating pro-oncogenic/anti-oncogenic genes or pathways, can prevent cancer incidence and inhibit cancer progression. In this review article, we briefly examine the possibility and the related molecular mechanisms on how TRZD intervention may increase the risk of cancer incidence and progression or whether TRZD has the potential to be used alone or in combination with other commonly used anti-cancer interventions (surgery, chemotherapy, radiation) to enhance the efficacy of cancer treatments.

## 2. Possible Anti-Cancer Effects of TRZD

**Figure 1:**
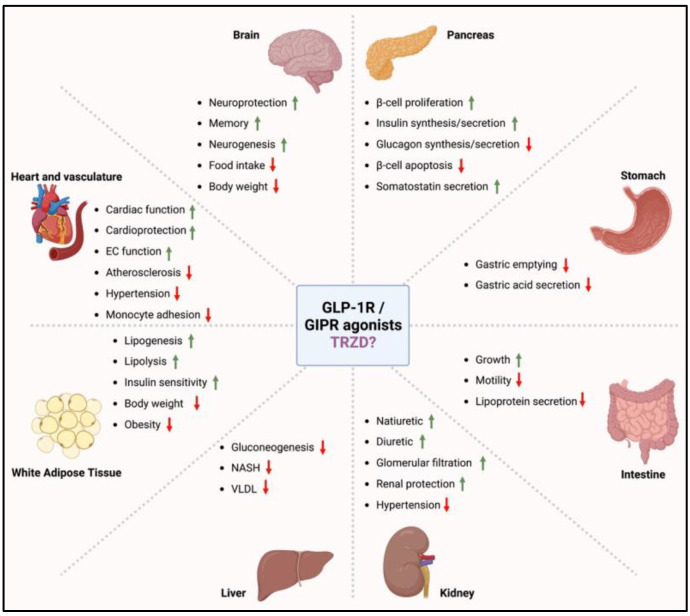
Possible target sites of GLP-1R/GIPR agonists and possibly TRZD. The incretin hormones GLP-1 and GIP secreted by the gastrointestinal tract have a wide array of target tissue that modulate various organ system functions. GLP-1R/GIPR agonists and possibly TRZD play several beneficial and protective functions in the various organ systems. The green upward arrow indicates in-crease/activation, while the red downward arrow indicates decrease/inhibition. EC = endothelial cell, NASH = non-alcoholic steatohepatitis and VLDL = very low-density lipoprotein. Created with Biorender.com (accessed on 14 September 2022).

Beyond their pleiotropic effects, the incretin mimetics or GLP-1R/GIPR agonists could be beneficial and protective to multiple organ systems (brain, pancreas, stomach, intestine, kidneys, liver, adipose tissue, heart, and vasculature) in many different aspects (Figure 1). While significantly decreasing blood glucose levels and increasing insulin sensitivity, TRZD treatment also supported a reduction in body weight. It reversed dyslipidemia in the direction of an improved cardiometabolic profile [26,27]. The GLP-1R/GIPR agonists can also regulate the function of the skeletal muscle, immune system, and brown adipose tissue [28,29].

In cancers, owing to the link between high blood glucose levels/diabetes and obesity to the incidence of cancer and its progression, the TRZD treatment-mediated decrease in blood glucose and body weight will most likely inhibit cancer growth and progression. Liragludtide (a GLP-1R agonist) treatment sensitized gemcitabine-resistant pancreatic cell line (PANC-1) to gemcitabine leading to inhibition of cell proliferation and activation of apoptosis [30]. Similar effects were observed in other gemcitabine-insensitive pancreatic cancer cell lines (MiaPaca-1 and MiaPaca-2) [30]. The ability of liraglutide to reverse chemoresistance was mediated by the increased expression of GLP-1R and decreased levels of NF-κB and muti-drug resistance protein ABCG2 in the treated cells [30]. Zhao et al. reported that the GLP-1R activation in response to liraglutide treatment in human pancreatic cancer cell lines and a mouse xenograft model was associated with inhibiting the pancreatic cancer growth and increased apoptosis via the inhibition of Akt and ERK1/2 signaling pathways [31]. Liraglutide inhibits cancer growth and metastasis of human pancreatic cancer cells in vitro and in vivo via the PI3K/Akt signaling [31].

Exenatide similarly exerted its antiproliferative effect on breast cancer cells via the inhibition of NF-κB nuclear translocation and suppression of oncogenic gene expression [32]. Exenatide also promoted apoptosis in breast cancer MCF-7 cells via the upregulation of various apoptotic and death-dependent genes [33]. In MCF-7 breast cancer cells, liraglutide inhibited cancer cell proliferation and promoted apoptosis via the inhibition of microRNA-27a [34]. Ligumsky et al. reported that exenatide attenuated the growth of two breast cancer xenografts (MDA-MB-231 and MDA-MB-468 cells) in athymic mice [35]. Treatment with either GLP-1 or exenatide increased cAMP levels associated with the activation of down-stream target CREB and up-regulation of CRE promoter transcription [35]. The authors concluded that cAMP seems to be a key mediator of exenatide’s anti-cancer activities [35].

Both exenatide and liraglutide inhibited cell proliferation and increased apoptotic cell death in prostate cancer cells in a dose-dependent manner via an increase in the ratio of pro-apoptotic Bax/anti-apoptotic Bcl-2 and activation of the p38 MAPK-dependent cell death pathway [36]. Exenatide treatment reportedly exerted antiproliferative effects in the prostate cancer cell lines (LNCap, PC3, and DU14) and attenuated the growth of LNCap-derived xenografts in athymic mice [37]. Results correlated with the significant downregulation of the tumor expression of P504S, Ki67, and phosphorylated ERK-MAPK [37]. A combination of exenatide and metformin showed an additive effect on the prostate tumor size reduction and inhibition of prostate cancer growth via inhibiting the ERK-MAPK pathway and the AMPK-related inhibition of the mTOR pathway [38].

In colon cancer cells, exenatide reduced the survival of murine colon cancer cell lines via increased levels of cAMP, which inhibits PI3K/Akt, GSK3, and ERK1/2 and activates pro-apoptotic caspase 3/7 [39]. Similar results were reported by Tong et al., using liraglutide in LOVO colon cancer cells [40]. Liraglutide suppressed the cell cycle, reduced cell proliferation, migration, and invasion, and induced apoptosis by inactivating the PI3K/Akt/mTOR signaling [40].

Exenatide treatment also protected against endometrial cancers via AMPK activation and inhibition of the mTOR pathway [41]. In another study, exenatide suppressed hyperglycemia-induced chemoresistance in human endometrial adenocarcinoma Ishikawa and HEC1B cells via ROS-mediated mitochondrial pathway [42]. Pro-apoptotic changes via increased Bax/Bcl-2, cytosolic cytochrome c, PARP, and acetylated-p53 were upregulated by cisplatin but downregulated by high-glucose treatment; however, these changes were reversed by exenatide [42]. Kanda et al., reported that liraglutide dose-dependently suppressed the proliferation of endometrial carcinoma Ishikawa cells [43]. These results were associated with drug-induced autophagy and phosphorylation of AMPK [43]. Moreover, increased GLP-1R expression correlated with positive estrogen receptor and progesterone receptor status and better progression-free survival [43]. Exenatide in ovarian cancer cells promotes apoptotic cell death by the activation of caspase 3/7 and inhibits cell proliferation by suppressing the PI3K/Akt pathway [44,45]. In a translation case-control study, exenatide significantly upregulated co-expressions of PSMA2 protein, and GLP-1 receptor in cervical cancer specimens gained from patients with diabetes were reduced by exenatide [46]. In addition, exenatide suppressed phospho-p65 and -IκB expressions in the NF-κB signaling in vitro and tumor volume and Ki67 expression in vivo [46].

The augmented expression of receptors for advanced glycation end-products (RAGE) and its ligands, advanced glycation end-products (AGEs), high-mobility group box 1 protein (HMGB1), and S100 group of proteins in obese and diabetic individuals was shown to increase the risk, growth, survival, progression, aggressiveness, chemoresistance, metastasis, and poor prognosis among patients suffering from cancers of the pancreas, breast, and colo-rectum [47,48]. The interaction between the AGEs and RAGEs modulates and supports several pro-oncogenic and anti-apoptotic pathways and influences the tumor microenvironment thus controlling the process of inflammation, fibrosis and tumor angiogenesis in growing and metastazing tumors [48,49]. Hence, targeting the AGE-RAGE axis should be beneficial in the effective treatment of several different cancers [47,48]. GLP-1, reportedly reduced the expression of RAGE, in vitro and in vivo, and played a protective role in preventing AGE-related adverse effects in diabetes [50]. In a rat model of non-alcoholic steatohepatitis (NASH), exenatide reportedly displayed hepato-protective effects by downregulating RAGE expression [51]. Similar results of a decrease in hyperglycemia-induced RAGE expression were observed in exenatide-treated rat ventricular myocytes [52]. The anti-inflammatory effect of exenatide treatment was evidenced by the attenuation of AGE-induced production of IL6 and TNFα in rat mesangial cells [53]. However, it remains unclear whether the effect of exenatide or other GLP-1R agonists in the AGE-RAGE axis translates to anti-neoplastic effects for the treatment of cancers.

Whether TRZD could have similar anti-cancer effects through the activation of the GLP-1R/GIPR is yet to be determined.

## 3. Possible Oncogenic Effects of TRZD

The effects of TRZD are mediated by the GLP-1 receptor (GLP-1R) and the GIP receptor (GIPR). Concerns regarding the pro-oncogenic effects of TRZD are centered on the potential carcinogenic properties and the related molecular pathways. These are mainly triggered by GLP-1R and GIPR agonists and DPP4 (dipeptidyl peptidase 4) inhibitor (inhibits DPP4 mediated GLP-1 and GLP-2 degradation, leading to sustained high levels of both GLP-1 and GLP-2) intervention, mainly on the pancreas and the thyroid.

GLP-1R expression in non-neoplastic tissues has been observed in the pancreatic islets, acini, stomach, duodenal Brunner’s gland, small and large intestinal myenteric plexus, lung and kidney vasculature, breast parenchyma, heart, brainstem, hypothalamus, neurohypophysis, and meninges [54]. Interestingly, the GLP-1R density/expression was significantly higher (overexpressed) in insulinomas (high expression in benign insulinomas and lesser expression in malignant/metastasizing insulinoma) and medullary thyroid carcinomas) and other endocrine, embryonal, and brain tumors [54]. In physiology, the species difference in the expression of GLP-1R is also noteworthy and will play a role in the effect GLP-1R agonists (such as TRZD) on cancers. Although the expression of GLP-1R was higher in the lungs of rats compared to the humans, mice thyroid medullary C-cells showed a significant expression of GLP-1R but were more or less absent in the human thyroid tissue [54,55].

GIPR is expressed in the pancreas, bones, brain, and adipose tissue [56,57]. GIPR was significantly higher in neuroendocrine tumors (NETs), such as functional pancreatic NETs (including insulinomas), gastrinomas, and non-functional pancreatic NETs, compared to non-neoplastic tissue [58]. Colorectal cancers (CRC) express GIPR, which may facilitate GIPR ligand binding and subsequent proliferation of CRC cells via the modulation of pathways that support cancer progression. GIP, as such, is overexpressed in obesity, which could partly explain the higher incidence of CRC in obese individuals [59].

### 3.1. In the Pancreas

Therapeutic intervention using GLP-1 receptor agonists, such as exenatide and liraglutide, was correlated to an increased risk of pancreatitis [60,61,62,63,64,65,66]. A study assessing the pancreatic safety of using sitagliptin, a DPP4 inhibitor, reported a small increase in the risk of developing pancreatitis [67]. A significant increase in pancreatitis was described by the US FDA advance event reporting system (FAERS) when using sitagliptin and exenatide in comparison to patients using other anti-diabetic drugs (rosiglitazone, glipizide, nateglinide, reparglinide) to control their blood glucose levels [68].

In the general population, having chronic pancreatitis, possibly due to an inherited (hereditary/familial) gene (PRSS1, SPINK1, CTRC, CASR, and CFTR) mutation, significantly increases the risk of developing pancreatic cancer by about 40% [69,70]. The sustained release of pro-inflammatory cytokines, increase in the levels of reactive oxygen species and related oxidative stress, the dysregulated cellular proliferation, as well as the intraductal pressure due to stenosis of the pancreatic duct(s) characteristic of chronic pancreatitis increases the risk of possible malignant transformation of the pancreas leading to pancreatic cancer (by approximately 26-fold), a diagnosis with a significantly high risk of an unfavorable prognosis [66,70,71,72,73]. Although it is not clear whether an episode of acute pancreatitis (such as those triggered by incretin mimetic intervention) could trigger a similar malignant transformation of the pancreatic tissues, it is worthwhile to educate the at-risk patients and monitor them for any symptoms/signs of pancreatitis.

Retrospective studies have reported that incretin mimetic administration was associated with an increased risk of pancreatic cancer [74]. The occurrence of pancreatic cancer was also more often reported among patients on a GLP-1 agonists-based treatment regimen [68]. A marked expansion in the exocrine and endocrine compartments of the pancreas with an increase in pancreatic mass augmented exocrine cell proliferation, and dysplasia was reported in diabetic patients on incretin mimetic therapy [75]. Evident α-cell hyperplasia and progressive glucagon-producing neuroendocrine tumors were observed in diabetic patients on incretin mimetic therapy [75].

**Figure 2:**
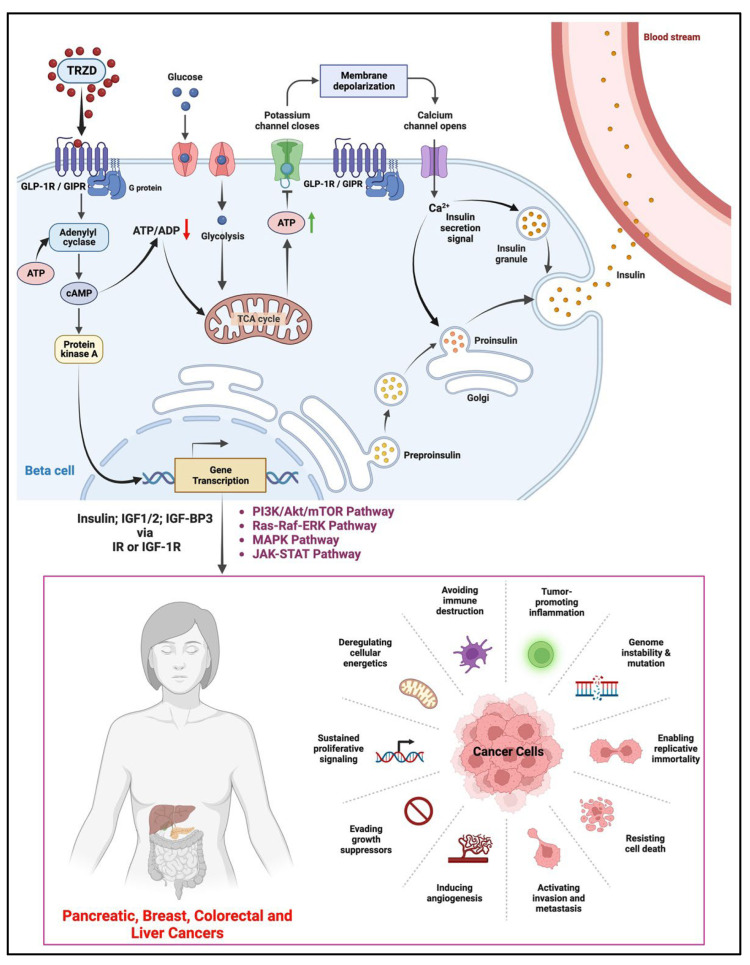
Possible pro-oncogenic effect of tirzepatide (TRZD) on the pancreas, breast, colorectal, and liver cancers. TRZD, possibly via GLP-1R or GIPR activation, leads to intracellular Ca^2+^-dependent release of insulin from the pancreatic β-cells. The GLP-1R activation can also lead to protein kinase A-dependent gene transcription leading to the synthesis and release of insulin-like growth factor 1 or 2 (IGF 1/2) and insulin-like growth factor binding protein 3 (IGF-BP3). Insulin and IGF 1/2 via the insulin receptor (IR) and the IGF receptor 1 (IGF-1R) in turn activate the PI3K/Akt/mTOR, Ras-Raf-ERK, MAPK, and JAK-STAT pathway, which supports several oncogenic and cancer progression traits such as resisting cell death, induction of angiogenesis, sustained proliferative signals, tumor inflammation, and activation of invasion and metastasis in target tissues (pancreas, breast, colorectum and liver). The black arrows indicate the pathway and flow of components involved in the pathway. The green upward arrow indicates an increase, while the red downward arrow indicates a decrease. Created with Biorender.com (accessed on 15 September 2022).

In the pancreatic β-cell, it is possible that TRZD treatment and subsequent GLP-1R or GIPR activation (Figure 2) via the adenylyl cyclase-cAMP-protein kinase A pathway activates the glycolytic and mitochondrial metabolism of glucose, in turn increasing intracellular ATP. The increase in the ATP/ADP leads to the closure of the plasma membrane K^+^ channels, eventually leading to β-cell depolarization, the subsequent opening of the voltage-gated Ca^2+^ channels, and the resultant influx of Ca^2+^ into the cell (the endoplasmic reticulum also releases Ca^2+^ in response to the initial elevation of cytosolic Ca^2+^). The increase in cytosolic Ca^2+^ triggers the release of insulin into the bloodstream. The activation of protein kinase A also, in turn, activates insulin gene transcription leading to the synthesis of insulin, insulin-like growth factors 1 and 2 (IGF 1 and 2), and insulin-like growth factor binding protein 3 (IGF-BP3), which via the insulin receptor or the insulin-like growth factor receptors activates the key pathways such as PI3K/Akt/mTOR, Ras-Raf-ERK, MAPK and JAK-STAT pathway in target cells [58,66]. The aberrant activation of these signaling pathways in an individual with a prior history of pancreatitis could significantly increase the risk of developing pancreatic cancer [76,77,78,79]. Insulin and insulin-like growth factors also activate these oncogenic pathways in other target tissues such as the breast, liver, and colorectum; thereby driving the progression of cancers by supporting cellular proliferation and growth, aberrant cancer metabolism, inhibition of cell death, invasion, and metastasis in these tissues [8,80,81].

### 3.2. In the Thyroid

Apart from the normal pancreatic cells, the GLP-1R and GIPR are expressed in the parafollicular of C-cells of the thyroid that secrete calcitonin [22,23]. Experimental evidence in rodents suggests a higher incidence of thyroid C-cell tumors in response to GLP-1 agonist intervention [22,23,73].

**Figure 3:**
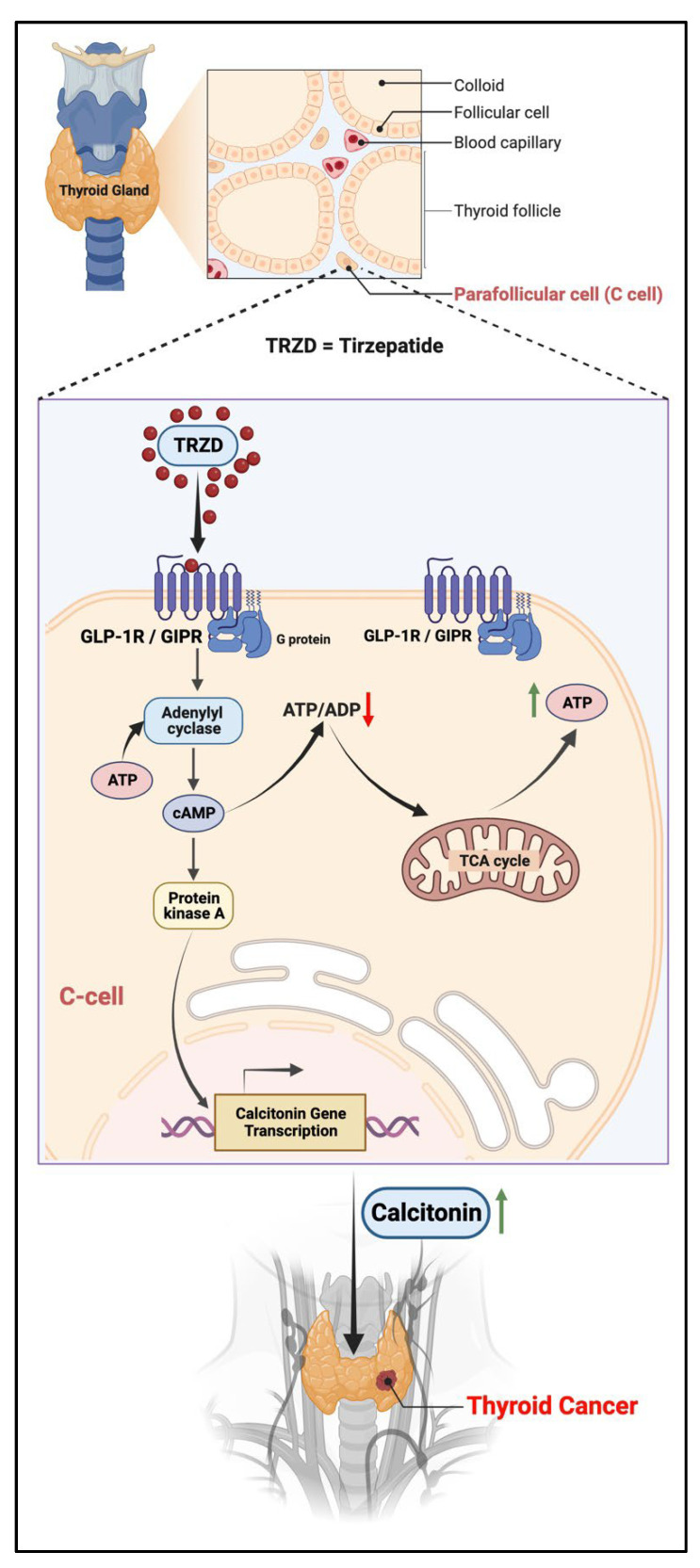
Possible pro-oncogenic effect of tirzepatide (TRZD) on the thyroid gland. TRZD via GLP-1R or GIPR activation leads to an increase in the intracellular levels of cAMP and protein kinase A activation, which drives the expression and secretion of calcitonin. The sustained activation of these receptors and release of calcitonin is accompanied by C-cell hyperplasia and transformation into C-cell adenomas and MTC. The black arrows indicate the pathway and flow of components involved in the pathway. The green upward arrow indicates an increase, while the red downward arrow indicates a decrease. Created with Biorender.com (accessed on 13 September 2022).

Anti-hyperglycemic incretin mimetics/GLP-1 analogs/agonists (such as liraglutide, exenatide, taspoglutide, and lixisenatide) caused a potent dose-dependent increase in cAMP levels and protein kinase A activation in the thyroid C-cells, leading to the activation of calcitonin gene expression, and thus triggered the release of calcitonin [22,23]. The sustained activation of these receptors and release of calcitonin is accompanied by C-cell hyperplasia and transformation into C-cell adenomas and MTC [22,73]. Treatment with TRZD (dual GLP-1R and GIPR agonist) could have a similar effect as the other incretin mimetic anti-diabetic drugs (Figure 3).

The effect of TRZD may vary depending on the species-related difference in the expression levels of the GLP-1R and GIPR. In primates and humans, the expression of GLP-1R in the C-cells of the normal thyroid is, however, significantly low or absent compared to that of the GLP-1R expression in thyroids of rodents [82,83]. Cell lines derived from rodent thyroid C-cells, when exposed to GLP-1 receptor agonists, lead to an elevation of cAMP levels and subsequent calcitonin release [23]. This effect of GLP-1 receptor agonists on cAMP levels and calcitonin secretion was not observed in cell lines derived from human thyroid C-cells, possibly due to the lower expression of GLP-1R on the human-derived cells [23]. Hence, the effect of TRZD or other GLP-1 agonists on calcitonin release and MTC may not be as pronounced in other primates and humans as in rodents. However, in rodents and humans, the expression of GLP-1R and GIPR was significantly higher in MTCs compared to normal thyroid tissue across the same species [82,84]. TRZD treatment thus may be a cause of concern if used in patients with MTC (or anyone in the family of the patient has had MTC) or if the patient has multiple MEN2, owing to the possibility of risk of developing and/or progression of MTCs in affected individuals [84].

## 4. Clinical Data and Trials

Clinical trials (ClincalTrials.gov, accessed on 12 October 2022); search keyword: tirzepatide) are underway to test and continue testing the efficacy of TRZD alone and in comparison with other anti-diabetic drugs for the treatment of type 2 diabetes, obesity, cardiovascular events, heart failure, and obstructive sleep apnea. The drug is also tested for adverse events in participants with impaired liver (hepatic insufficiency and non-alcoholic steatohepatitis) and renal function (renal insufficiency and end-stage renal disease).

Data from ClincalTrials.gov (accessed on 12 October 2022) includes a list of 29 (search performed on 12 October 2022; using keywords diabetes and tirzepatide) tirzepatide administration-based clinical trials concerning/specific to diabetes (Table 1). In total, 16 trials out of the 29 were completed. Moreover, 13 tirzepatide and diabetes-related clinical trials are currently active, out of which 4 are active and recruiting patients, whereas two clinical trials have not started recruiting subjects for the study (Table 1).

On the other hand, a search using the keywords obesity and tirzepatide yielded 14 tirzepatide administration-based clinical trials related to/specific to obesity (Table 1). Though 2 trials out of the 14 are completed, 12 tirzepatide and obesity-related clinical trials are currently active, out of which 4 are active and recruiting patients, and 2 clinical trials have not started recruiting subjects for the study (Table 1).

A search using the keywords cancer and tirzepatide did not yield any results (Table 1).

## 5. Conclusions and Future Perspective

TRZD is considered a novel glucose-lowering drug since it is the first and only dual-acting GLP-1R/GIPR agonist that FDA has approved for the treatment of diabetes. Furthermore, the recommended anti-hyperglycemic dosage of TRZD significantly promoted weight loss and may be used in the treatment of obesity. Reports suggest that other GLP-1R/GIPR agonists, besides their glucose-lowering effect, can be valuable for the prevention and treatment of cardiovascular diseases, metabolic diseases, and neuro- and endocrine disorders owing to their vast array of multi-faceted regulatory effects on the different organ systems. Furthermore, GLP-1R/GIPR agonists were found to have several anti-neoplastic effects in different cancers. Basic and clinical research studies are warranted to ascertain whether TRZD, a dual-acting GLP-1R/GIPR agonist, can bring about similar protective and beneficial effects, as reported in the case of other GLP-1R/GIPR agonists.

Although controversial, TRZD treatment may likely promote/support cancers of the pancreas, thyroid, breast, liver, and colon. However, with the currently available short-term data, it is difficult to ascertain whether the benefits of using tirzepatide for treating diabetes and/or obesity could outweigh its risks. For instance, although it is established that chronic pancreatitis increases the risk of pancreatic cancers, this may not hold in cases of acute pancreatitis such as those caused by the GLP-1/GIP analogs [73]. Based on the data on known mutation rates, it is estimated that it may take over 20 years for normal pancreatic duct cells to transform and grow into a pancreatic carcinoma with metastatic capacity and be diagnosed due to its clinical symptoms [73]. Once diagnosed with diabetes, the individual may be maintained on the anti-diabetic treatment regimen for the rest of his/her life. If the individual is on a GLP-1R/GIPR agonist or TRZD, the long-term exposure to these drugs might accelerate the progression of malignant disease in target tissues. Hence, the study subjects involved in the clinical trials using TRZD need to be followed up and monitored for several years, which will help acquire reliable data on the risks for the malignant disease since the development of cancer can most likely only be influenced over a long period.

**Figure 4:**
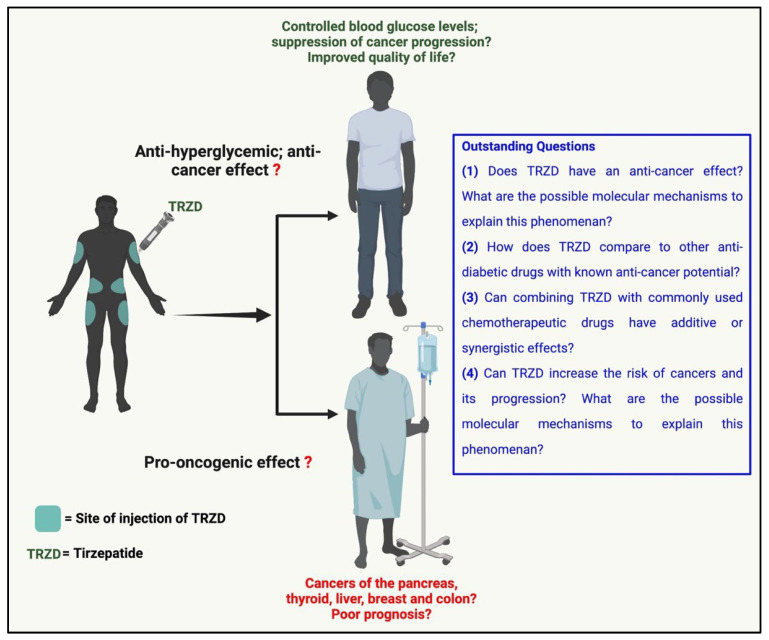
Outstanding questions regarding the use of tirzepatide (TRZD) in diabetic/obese or diabetic-/obese-cancer patients. Created with Biorender.com (accessed on 15 September 2022).

Nonetheless, the safety profile of tirzepatide must be precisely monitored, and any serious adverse events (SAEs) should be strictly reported without bias. The completed and currently active clinical trials must educate their participants to identify/recognize and report any adverse event and carry out a long-term follow-up of their participants to identify any SAEs. Basic scientists must come forward to test tirzepatide for its potential cancer-promoting or cancer therapeutic effects. Clinical trials should address the efficacy and safety of using tirzepatide in diabetic non-cancer and cancer patients regarding the possibility of linking it to cancer incidence and progression. There are several outstanding questions (Figure 4) regarding TRZD’s effect on cancer. The pro-oncogenic or anti-cancer effect of TRZD and the possible molecular mechanisms involved are yet to be determined. It is still unknown how TRZD compares with other anti-diabetic drugs with known anti-neoplastic abilities. Whether a combination of TRZD and routinely used chemotherapeutic drugs can efficiently treat cancers is yet to be answered.

## Figures and Tables

**Table 1:** Status of tirzepatide administration-related clinical trials. (Search keywords: Diabetes/Obesity/Cancer + Tirzepatide) (https://clinicaltrials.gov/ (accessed on 12 October 2022).

Serial No.	Condition	Total Registered Trials	Completed	Active, Not Recruiting	Active, Not Yet Recruiting	Active, Recruiting
1	Diabetes	29	16	7	2	4
2	Obesity	14	2	6	2	4
3	Cancers	-	-	-	-	-

## Data Availability

Not applicable.

## Abbreviations

AGEs	Advanced glycation end-products
Akt	Protein kinase B
AMP	Adenosine monophosphate
ATP	Adenosine triphosphate
cAMP	3′,5′-cyclic AMP
DPP4	Dipeptidyl peptidase 4
ERK	Extracellular signal-regulated kinase
FAERS	FDA advance event reporting system
FDA	Food and drug administration
GIP	GIP receptor
GIP	Glucose-dependent insulinotropic polypeptide/Gastric inhibitory peptide
GLP-1	Glucagon-like peptide 1
GLP-1R	GLP-1 receptor
IGF-1R	IGF-1 receptor
IGF-BP3	Insulin-like growth factor binding protein 3
IGF1/2	Insulin-like growth factor 1/2
JAK	Janus kinase
MAPK	Mitogen activated protein kinase
MEN2	Multiple endocrine neoplasia syndrome type 2
MTC	Medullary thyroid cancer/carcinoma
mTOR	mammalian target of rapamycin
NF-κB	Nuclear factor kappa-B
PARP	Poly (ADP-ribose) polymerase
PI3K	Phosphoinositide 3-kinase
PSMA2	Proteasome alpha 2 subunit
RAGEs	Receptors of AGEs
SGLT2	Sodium glucose co-transporter 2
STAT	Signal Transducer and Activator of Transcription
TRZD	Tirzepatide
WHO	World Health Organization
